# Supplementary material for: Mapping global dynamics of benchmark creation and saturation in artificial intelligence
Source: Nat Commun. 2022 Nov 10;13:6793. doi: 10.1038/s41467-022-34591-0 (PMC9649641; doi:10.1038/s41467-022-34591-0)
Supplement: Supplementary file 1 — Supplementary Information [file 41467_2022_34591_MOESM1_ESM.pdf]

Mapping global dynamics of benchmark creation and saturation in artificial intelligence

Supplementary Information

Supplementary Figure 1: Global SOTA improvement map for computer vision. Anchors (vertical dashes) and only multiple dot trajectories shown in the plot.
Supplementary Figure 2: AI benchmark lifecycle map for computer vision.
Supplementary Figure 3: Number of active benchmarks vs. number of benchmarks reporting novel SOTA results over time for computer vision tasks.

High definition images of all figures are available here:
https://drive.google.com/drive/folders/12aA8t49sp7hxqVZJdWCrh-gvVolxtYeh

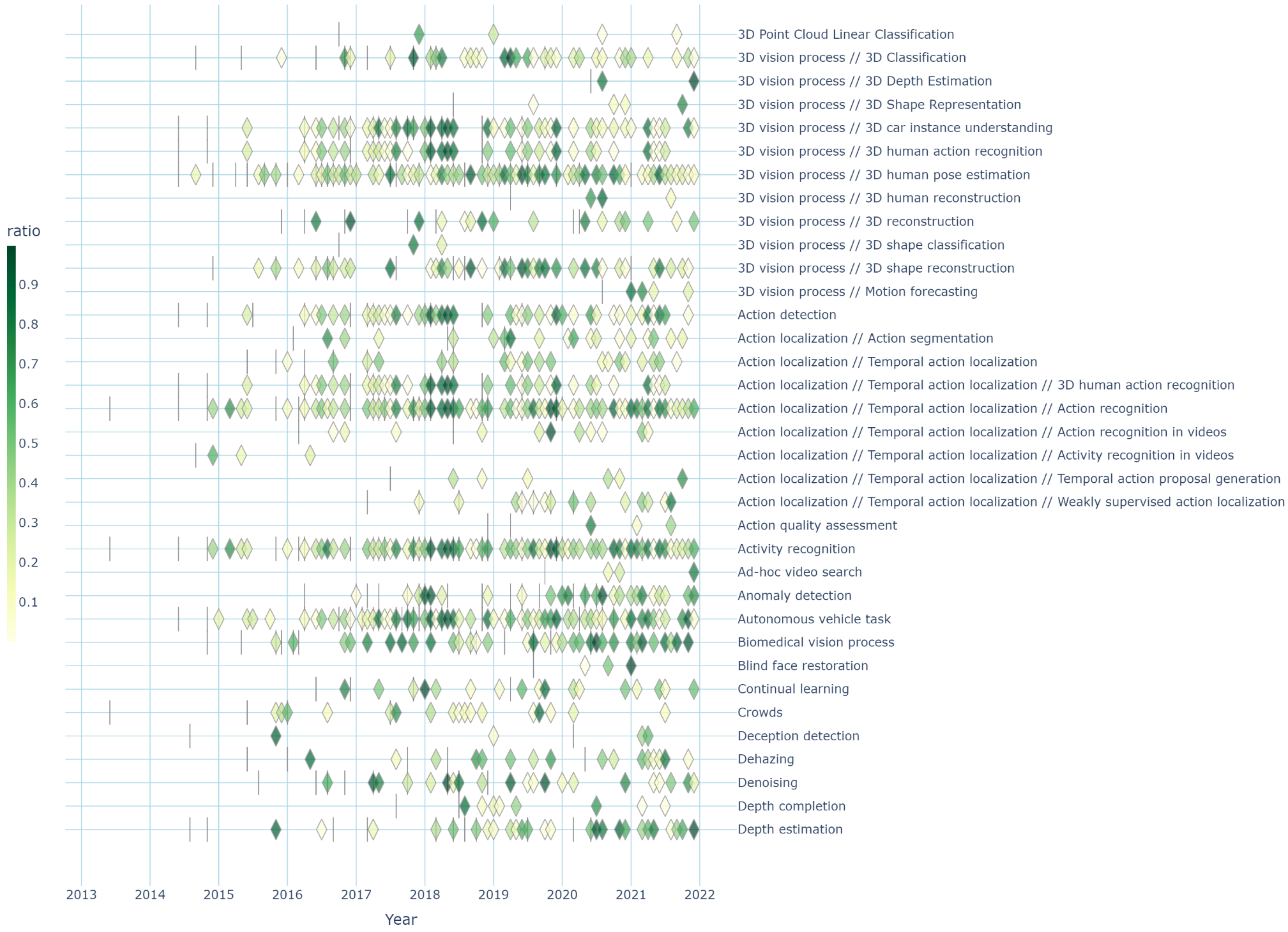

Supplementary Figure 1 (continued on following pages): **Global SOTA improvement map for computer vision.**

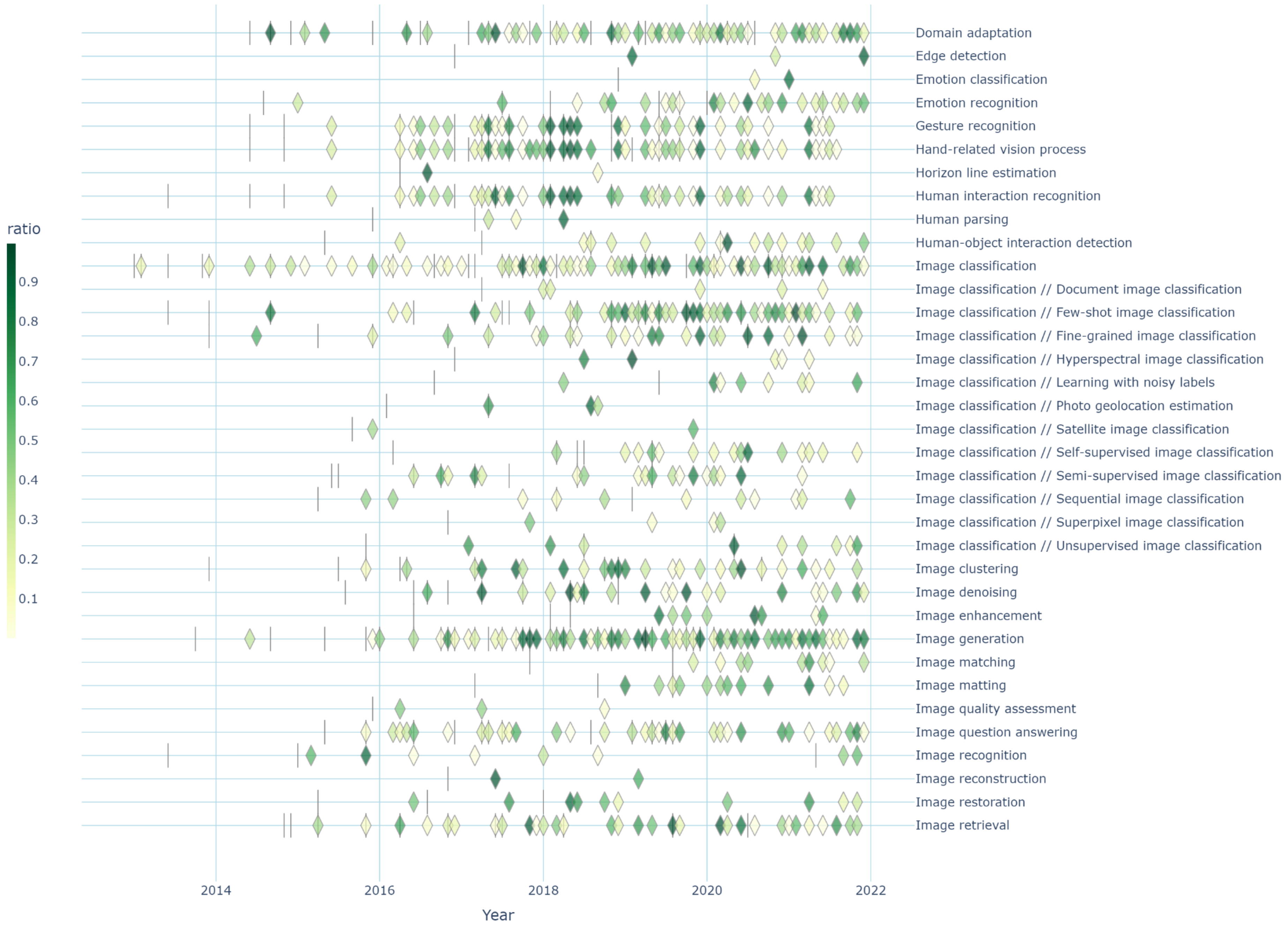

Supplementary Figure 1 (continued): **Global SOTA improvement map for computer vision.**

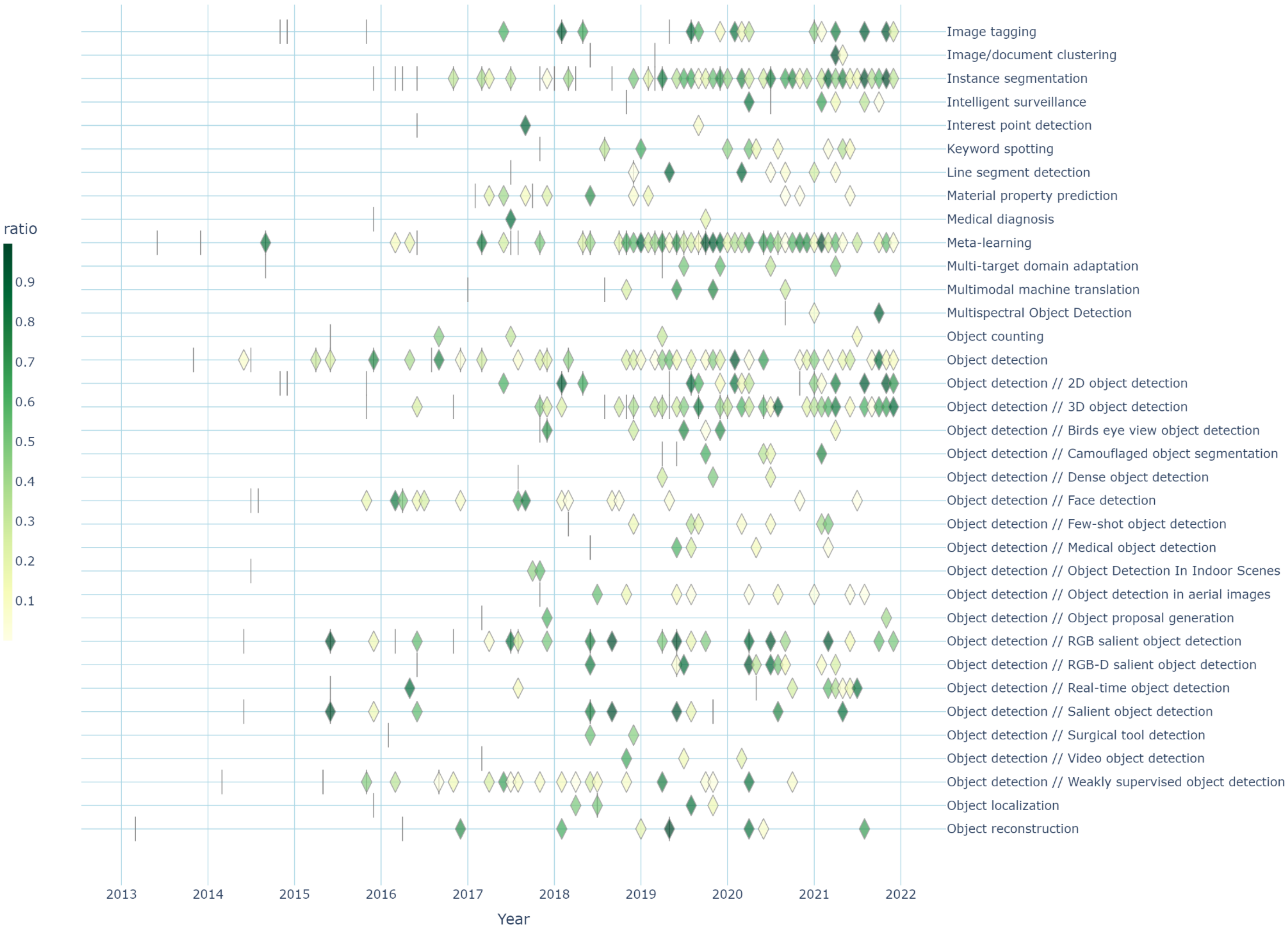

Supplementary Figure 1 (continued): **Global SOTA improvement map for computer vision.**

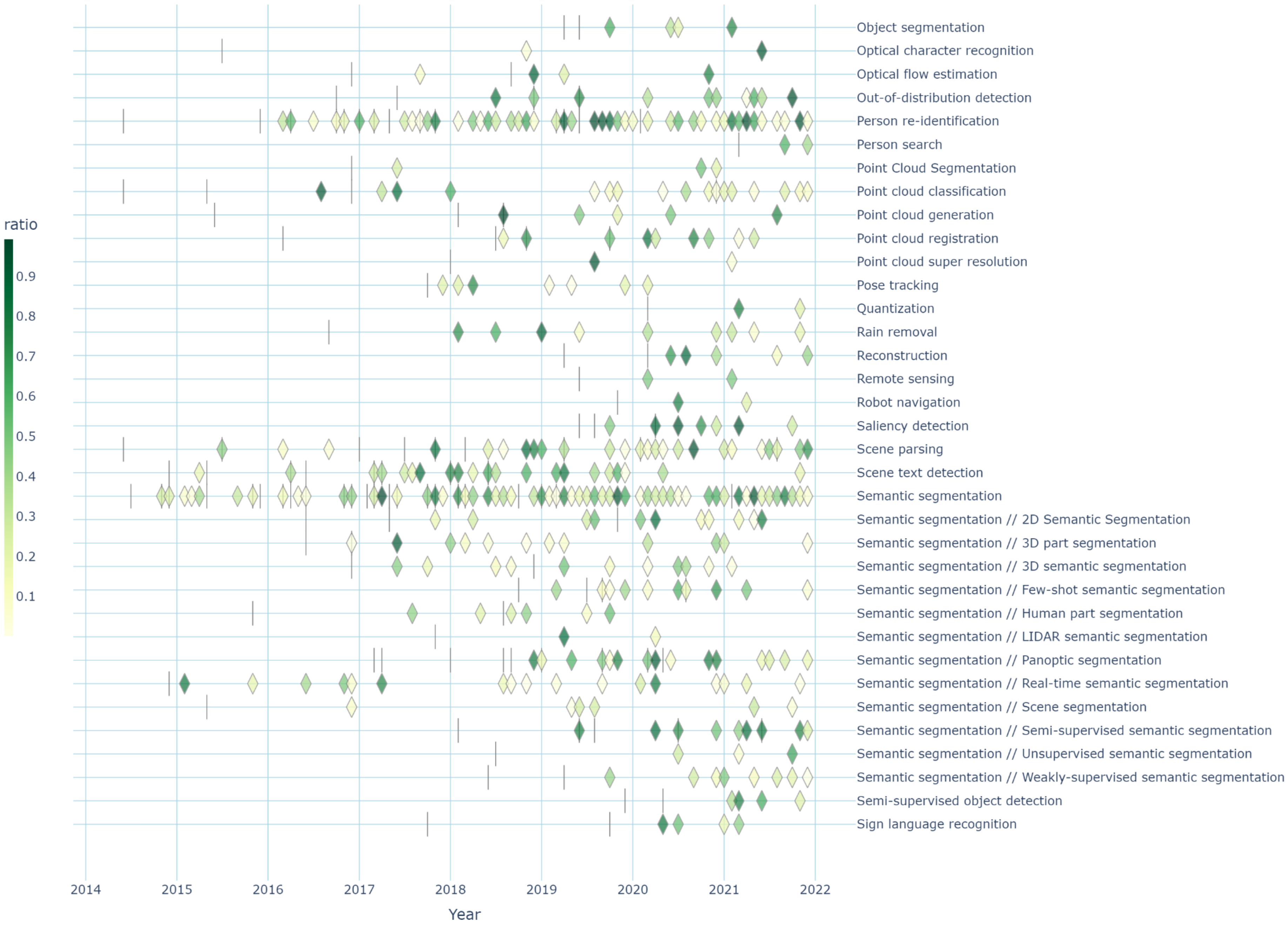

Supplementary Figure 1 (continued): **Global SOTA improvement map for computer vision.**

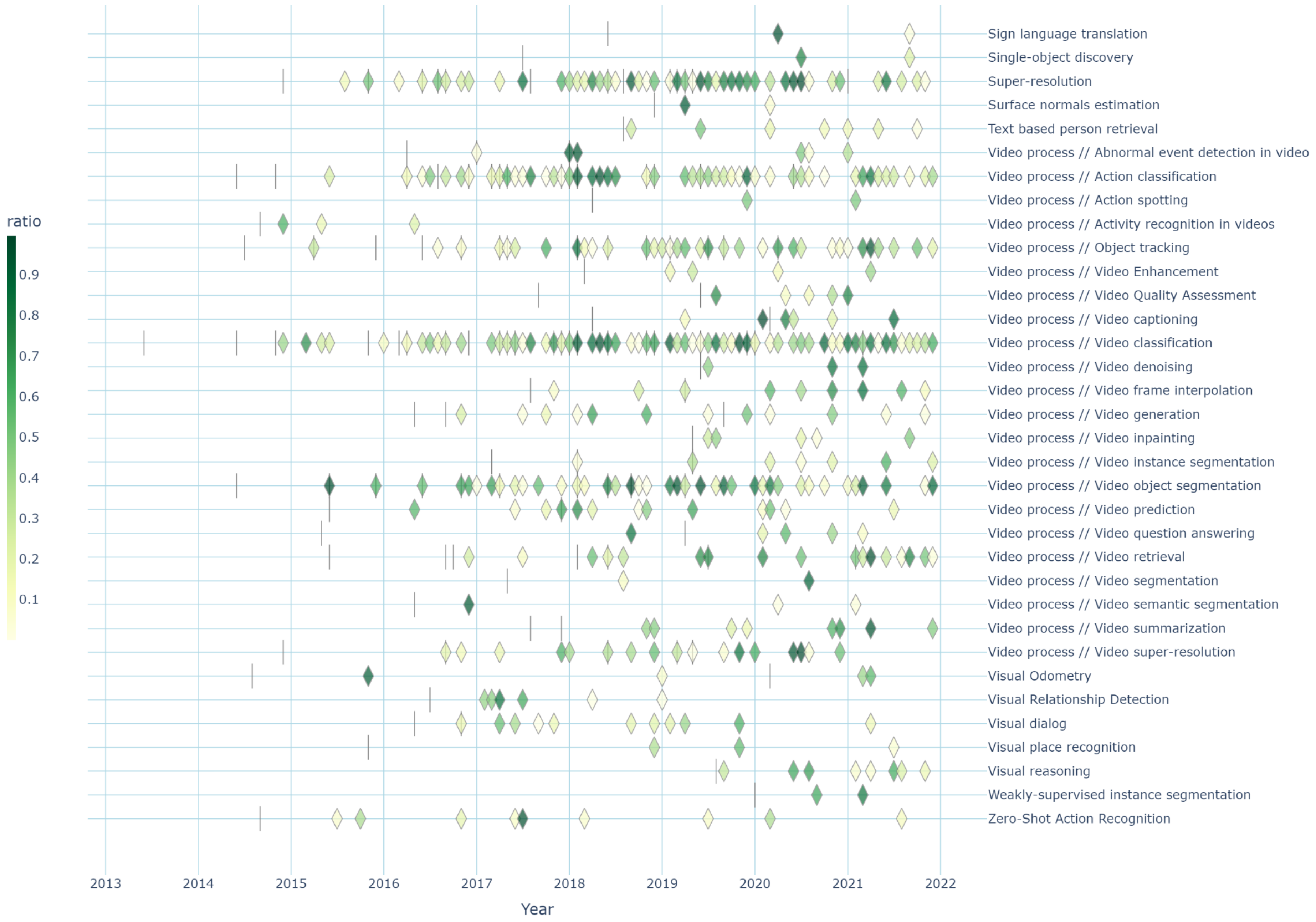

Supplementary Figure 1 (continued): **Global SOTA improvement map for computer vision.**

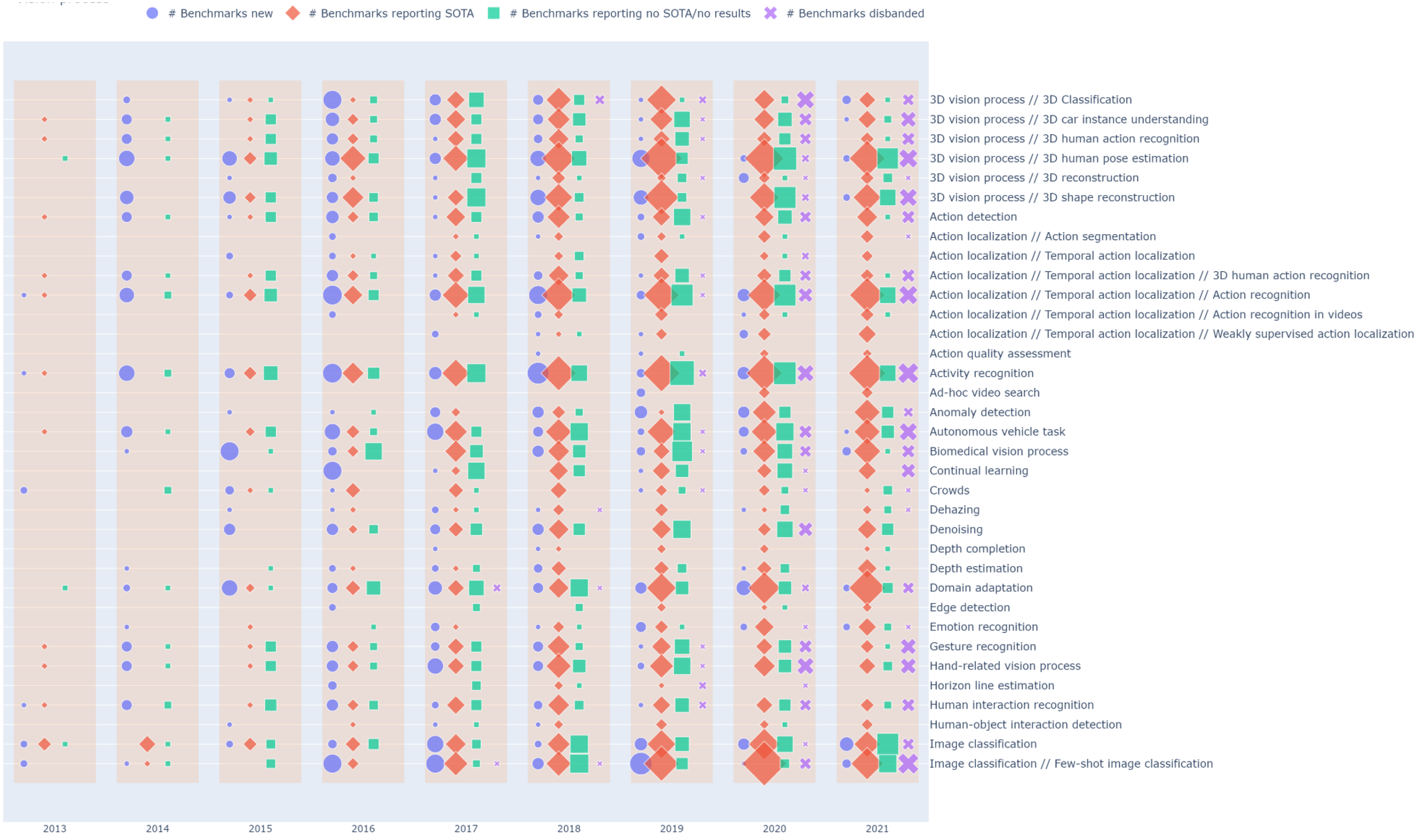

Supplementary Figure 2 (continued on following pages): **AI benchmark lifecycle map for computer vision.**

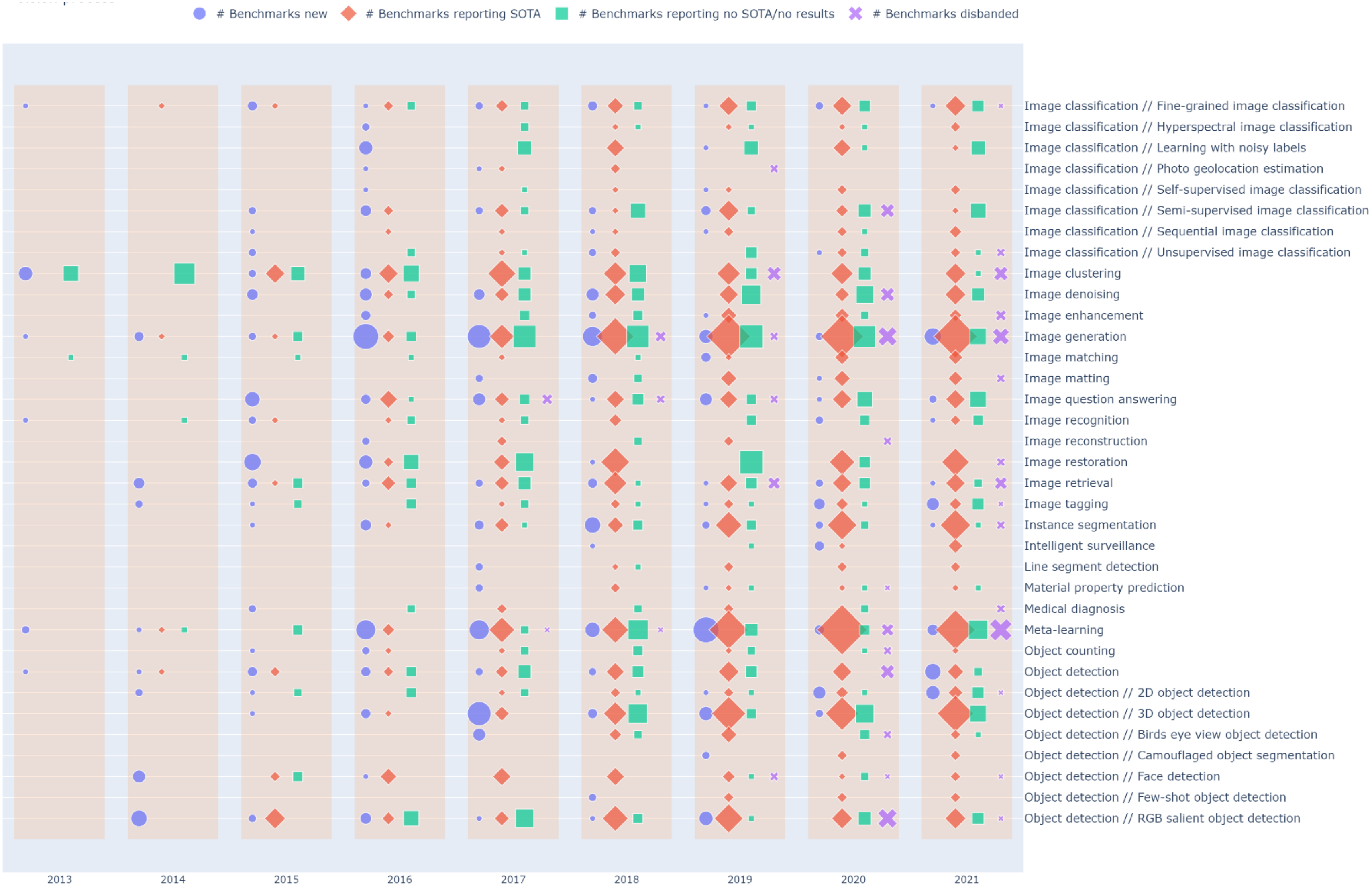

Supplementary Figure 2 (continued): **AI benchmark lifecycle map for computer vision.**

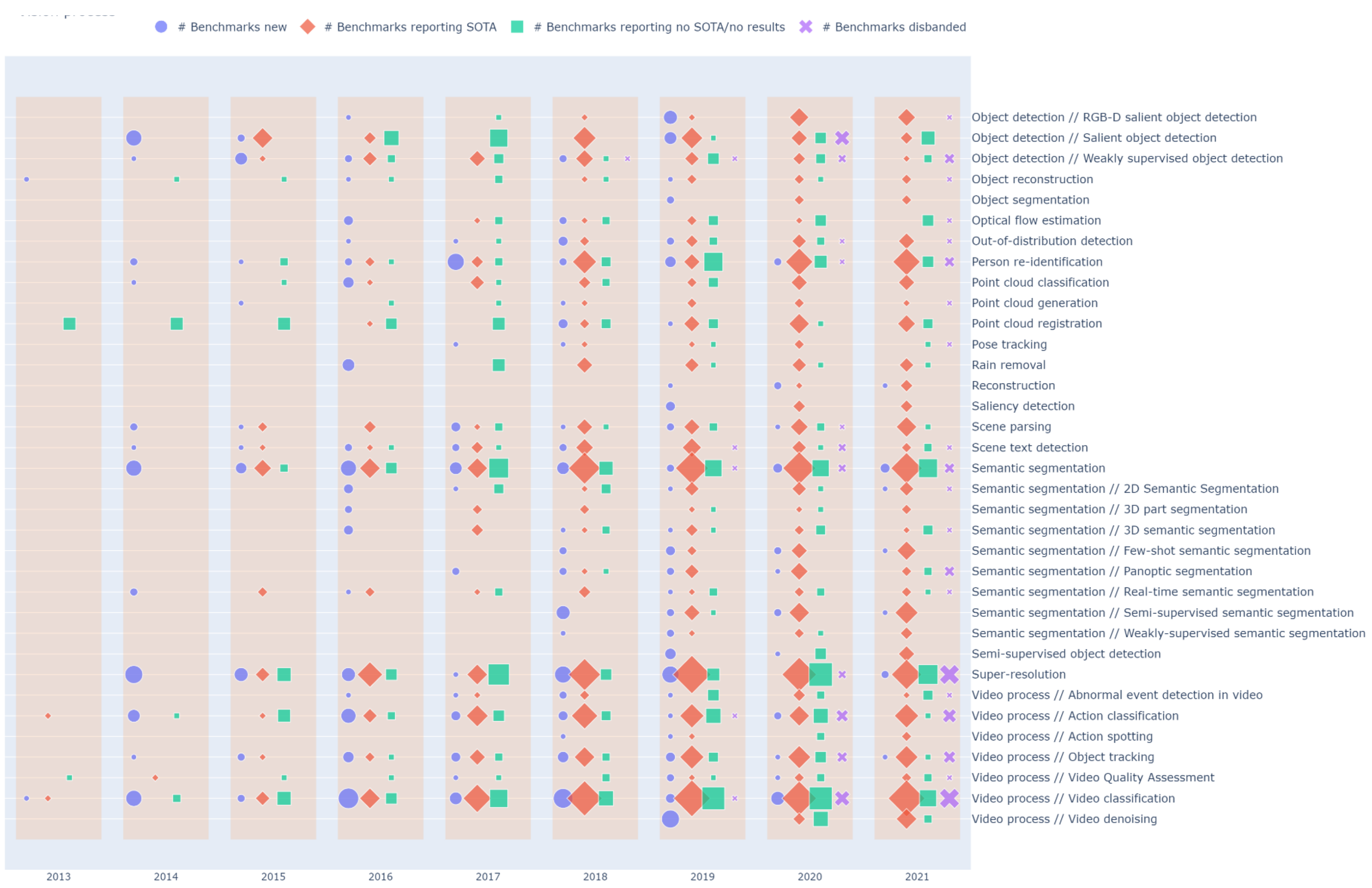

Supplementary Figure 2 (continued): **AI benchmark lifecycle map for computer vision.**

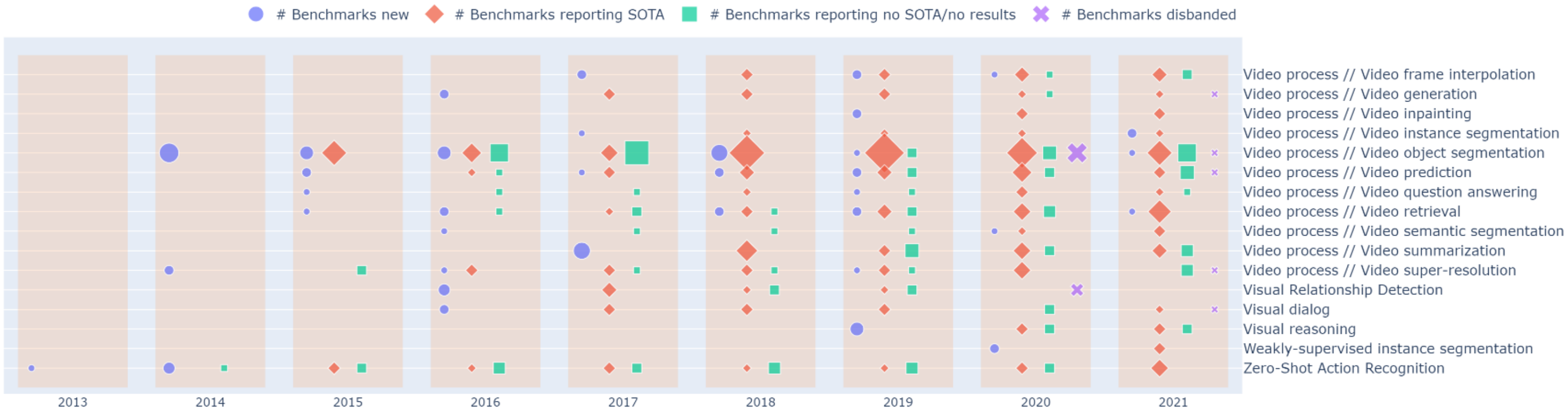

Supplementary Figure 2 (continued): **AI benchmark lifecycle map for computer vision.**

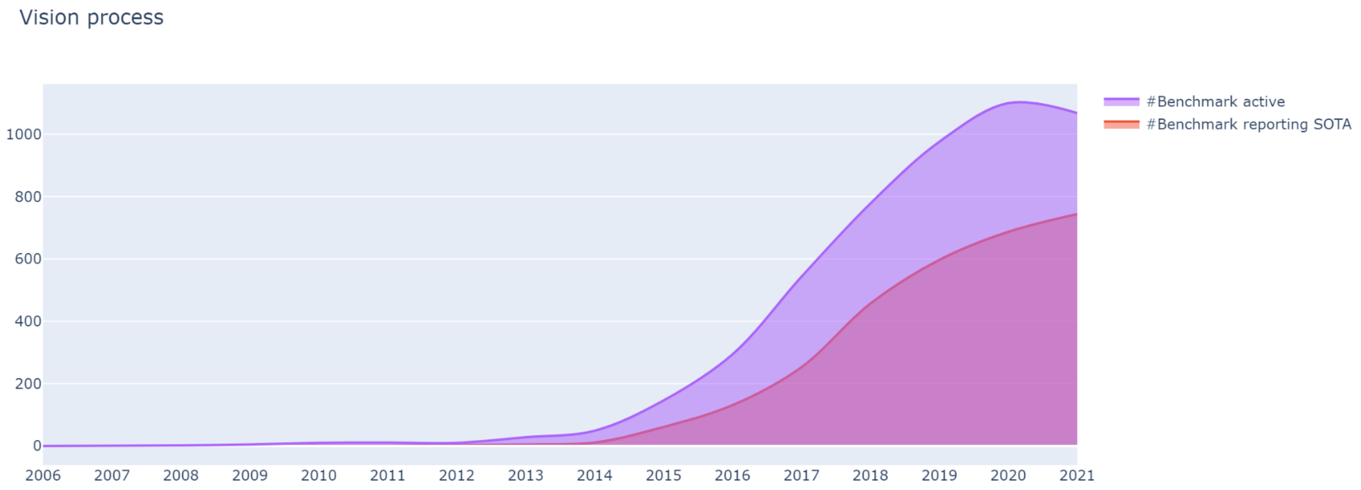

Supplementary Figure 3: **Number of active benchmarks vs. number of benchmarks reporting novel SOTA results over time for computer vision tasks.**
