# Supplementary material for: Mapping global dynamics of benchmark creation and saturation in artificial intelligence
Source: Nat Commun. 2022 Nov 10;13:6793. doi: 10.1038/s41467-022-34591-0 (PMC9649641; doi:10.1038/s41467-022-34591-0)
Supplement: Supplementary file 11 — Reporting Summary [file 41467_2022_34591_MOESM11_ESM.pdf]

nature portfolio

Corresponding author(s): Matthias Samwald

Last updated by author(s): Oct 12, 2022

Reporting Summary

Nature Portfolio wishes to improve the reproducibility of the work that we publish. This form provides structure for consistency and transparency in reporting. For further information on Nature Portfolio policies, see our Editorial Policies and the Editorial Policy Checklist.

Statistics

For all statistical analyses, confirm that the following items are present in the figure legend, table legend, main text, or Methods section.

n/a	Confirmed	
☐	☒	The exact sample size (*n*) for each experimental group/condition, given as a discrete number and unit of measurement
☐	☒	A statement on whether measurements were taken from distinct samples or whether the same sample was measured repeatedly
☐	☒	The statistical test(s) used AND whether they are one- or two-sided<br>*Only common tests should be described solely by name; describe more complex techniques in the Methods section.*
☐	☒	A description of all covariates tested
☐	☒	A description of any assumptions or corrections, such as tests of normality and adjustment for multiple comparisons
☒	☐	A full description of the statistical parameters including central tendency (e.g. means) or other basic estimates (e.g. regression coefficient) AND variation (e.g. standard deviation) or associated estimates of uncertainty (e.g. confidence intervals)
☐	☒	For null hypothesis testing, the test statistic (e.g. *F*, *t*, *r*) with confidence intervals, effect sizes, degrees of freedom and *P* value noted<br>*Give P values as exact values whenever suitable.*
☒	☐	For Bayesian analysis, information on the choice of priors and Markov chain Monte Carlo settings
☐	☒	For hierarchical and complex designs, identification of the appropriate level for tests and full reporting of outcomes
☒	☐	Estimates of effect sizes (e.g. Cohen's *d*, Pearson's *r*), indicating how they were calculated

Our web collection on statistics for biologists contains articles on many of the points above.

Software and code

Policy information about availability of computer code

Data collection	No software was used for data collection.
Data analysis	For the creation of the Intelligence Task Ontology and Knowledge Graph / ITO, we utilized the official data dump of the Papers With Code repository (data export date 2022/07/30) and devised Python scripts to convert the data into the Web Ontology Language (OWL) format 22. We conducted extensive manual curation of the task class hierarchy and the performance metric property hierarchy in the collaborative ontology editing environment WebProtége (version 4.0.2).<br>Data from ITO was loaded into the high-performance graph database BlazeGraph (version 2.1.6, blazegraph.com) and queried using the graph query language SPARQL. For data manipulation, we used Pandas (version 1.2.4) for manipulating large data frames and Numpy (version 1.20.3) for numeric calculations (such as average). Plotly (version 4.14.3) was used for data visualization in order to create trajectory plots and state of the art curves on Jupyter™ Notebooks (version 6.4.0) running Python (version 3.9.5). We created an interactive Global Map of Artificial Intelligence Benchmark Dynamics using the graphical library Plotly.express (version 5.10, plotly.com/python/plotly-express) in dedicated Jupyter notebooks using Python (see https://github.com/OpenBioLink/ITO/tree/master/notebooks)<br>For the creation of SOMs we used the Python library MiniSom (version 2.3.0, github.com/JustGlowing/minisom).

For manuscripts utilizing custom algorithms or software that are central to the research but not yet described in published literature, software must be made available to editors and reviewers. We strongly encourage code deposition in a community repository (e.g. GitHub). See the Nature Portfolio guidelines for submitting code & software for further information.

Data

Policy information about availability of data

All manuscripts must include a data availability statement. This statement should provide the following information, where applicable:

- Accession codes, unique identifiers, or web links for publicly available datasets
- A description of any restrictions on data availability
- For clinical datasets or third party data, please ensure that the statement adheres to our policy

Source data are provided with this paper. Benchmark data was downloaded from the Papers With Code repository available at https://github.com/paperswithcode/paperswithcode-data (export date 2022/07/30). The curated knowledge graph underlying our analyses is deposited online at https://doi.org/10.5281/zenodo.7097305. Supplementary data for the manuscript are deposited online at https://doi.org/10.5281/zenodo.7110147.

Human research participants

Policy information about studies involving human research participants and Sex and Gender in Research.

Reporting on sex and gender	N/A
Population characteristics	N/A
Recruitment	N/A
Ethics oversight	N/A

Note that full information on the approval of the study protocol must also be provided in the manuscript.

Field-specific reporting

Please select the one below that is the best fit for your research. If you are not sure, read the appropriate sections before making your selection.

☒ Life sciences ☐ Behavioural & social sciences ☐ Ecological, evolutionary & environmental sciences

For a reference copy of the document with all sections, see nature.com/documents/nr-reporting-summary-flat.pdf

Life sciences study design

All studies must disclose on these points even when the disclosure is negative.

Sample size	N/A
Data exclusions	No data were excluded from the analysis.
Replication	Reproduciibility of results is ensured by making all underlying data and analytical code openly available.
Randomization	The study is not an intervention study, but a descriptive analysis of an existing dataset. Randomization does not apply.
Blinding	The study is not an intervention study, but a descriptive analysis of an existing dataset. Blinding does not apply.

Reporting for specific materials, systems and methods

We require information from authors about some types of materials, experimental systems and methods used in many studies. Here, indicate whether each material, system or method listed is relevant to your study. If you are not sure if a list item applies to your research, read the appropriate section before selecting a response.

Materials & experimental systems

n/a	Involved in the study
☒	☐ Antibodies
☒	☐ Eukaryotic cell lines
☒	☐ Palaeontology and archaeology
☒	☐ Animals and other organisms
☒	☐ Clinical data
☒	☐ Dual use research of concern

Methods

n/a	Involved in the study
☒	☐ ChIP-seq
☒	☐ Flow cytometry
☒	☐ MRI-based neuroimaging
